# Are developmental shifts the main driver of phenotypic evolution in *Diplodus* spp. (Perciformes: Sparidae)?

**DOI:** 10.1186/s12862-019-1424-1

**Published:** 2019-05-21

**Authors:** Paolo Colangelo, Daniele Ventura, Paolo Piras, Jacopo Pagani Guazzugli Bonaiuti, Giandomenico Ardizzone

**Affiliations:** 1 Research Institute on Terrestrial Ecosystems, National Research Council, Via Salaria km 29.300, 00015 Monterotondo, Rome Italy; 2 Department of Biology and Biotechnologies “Charles Darwin”, Sapienza University, Via Borelli 50, 00161 Rome, Italy; 3 Department of Environmental Biology, Sapienza University, Rome, Italy; 4 Department of Cardiovascular Respiratory Nephrologic and Geriatric Sciences, Sapienza University, Rome, Italy

**Keywords:** Evo-devo, Morphological divergence, Ontogenesis, Evolutionary trajectory, Mediterranean Sea, *Diplodus*

## Abstract

**Background:**

Sparid fishes of the genus *Diplodus* show a complex life history. Juveniles have adaptations well suited to life in the water column. When fishes recruit into the adult population, individuals develop a radically differentiated shape that reflects their adaptation to the new benthic environment typical of the adult.

A comparative analysis of ontogenetic trajectories was performed to assess the presence of divergence in the developmental pattern**.** By using a geometric morphometric approach, we investigated the pattern of shape variation across ontogenetic stages that span from early settlement to the adult stage in four species of the genus *Diplodus*. Landmarks were collected on the whole body of fishes to quantify the phenotypic variation along two well defined life stages, i.e. juvenile and adult.

A comparative analysis of ontogenetic trajectories was performed to assess the presence of divergence in the developmental pattern. Subsequently, we investigated the patterns of integration and modularity as proxy for the alteration of the developmental processes. This have allowed to give an insight in morphological developmental patterns across ecologically and ontogenetically differentiated life stages and to investigate the process leading to the adult shape.

**Result:**

Our results suggest that the origin of morphological novelties in *Diplodus* spp. arise from shifts of the ontogenetic trajectories during development. During the settlement phase, the juveniles’ morphological shapes converge towards similar regions of the morphospace. When the four species approach the transition between settlement and recruitment, we observe the lowest level of inter- and intra-specific disparity. After this transition we detect an abrupt shift of ontogenetic trajectories, i.e. the path taken by species during development, that led to highly divergent adult phenotypes.

**Discussion:**

We suggest that the evolution of new ecomorphologies, better suited to exploit different niches (pelagic vs. benthonic) and reduce inter-specific competition in *Diplodus* spp., are related to the shift in the ontogenetic trajectory that in turn is associated to changes in modularity and integration pattern.

**Electronic supplementary material:**

The online version of this article (10.1186/s12862-019-1424-1) contains supplementary material, which is available to authorized users.

## Background

Understating how developmental processes influence the origin of phenotypic novelties is a central task of evolutionary biology. According to the Darwinian Theory, morphological differences observed between species can be explained through natural selection on heritable changes in phenotype [1]. Natural selection can gradually promote shape modifications with an adaptive significance, increasing the fitness of organisms in a specific ecological niche [1]. The neo-Darwinian synthesis predicts that the observed morphological differences arise by a progressive accumulation of gene mutations in partially isolated populations that are successively fixed by the evolution of barrier to geneflow, hence speciation [2–4]. However, while phenotypic variation is often assumed to be gradual, adaptive and molded by natural selection, additional mechanisms occurring during development, could play important roles in triggering and constraining morphological changes [5, 6]. The neo-Darwinian synthesis shows a rather limited consideration of developmental constraints and epigenetic interactions during growth as a source of morphological variation [7–9]. Development is a critical process whose abrupt alteration in many cases would lead to an unfit adult phenotype. It is thus reasonable to consider the ontogenetic processes as highly conservative so that closely related species are expected to show no or little differences in the development process, as a modification of the developmental program would involve alterations of single regulatory genes or of the entire developmental gene network still producing fit individuals. In this framework the changes in structure and function across species are expected to be the result of a continuous gradual processes with little influence on developmental processes [7]. However, in spite of the fact that development is known to act as constraint on phenotypic expression, there is accumulating evidence that it can act also as a driver of evolutionary change [10, 11]. From an evolutionary point of view we could argue that if we observe ecomorphological differences (i.e. morphological changes with specific ecological and adaptive significance) associated to ontogenetic trajectory shifts, these differences may underlie an alteration of developmental pathways [12]. In turn, these developmental alterations could represent the main driver of the evolution of new phenotypic adaptations. However, the evolutionary mechanisms that cause ontogenetic trajectories to shift and their impact on phenotypic diversity it is still debated [13–17]. Hence, it is important to accumulate new evidence on how changes in the developmental pathway may contribute to explain the origin of biological diversity.

Demersal fishes, which have a bipartite life history, with ontogenetically distinct life stages and adaptations (juveniles live in the water column feeding mostly on plankton whereas adults live and feed on or near the bottom), offer a unique opportunity to study the evolutionary pattern behind the appearance of ecomorphological novelties and their adaptive significance. Particularly, demersal fish species of the genus *Diplodus* show a remarkably high diversity of morphologies among species and between morphs belonging to different life stages (juveniles and adults). Juveniles of these species occur in benthic inshore habitats, are gregarious and can share the same nursery grounds [18]. Ventura et al. [19] found a clear correlation between trophic preferences and the morphology of the feeding apparatus among juveniles of these species, suggesting that selection acts to guarantee the adaptation to specific trophic niches in the earliest post-embryonic stages, reducing potential inter-specific competition. Subsequently, *Diplodus* spp. undergo new important crucial ontogenetic niche shifts when juveniles recruit into the adult population reaching a very distinct morphology [20]. In temperate waters, adults of these species dominate fish assemblages among rocky, sandy and seagrass infralittoral bottoms. The adult life stage takes place in open deep waters (up to 150 m) and show clear morphological inter-specific differences linked to trophic and micro-habitat preferences [21, 22]. The mechanism leading to the abrupt morphological change between juveniles and adults, accompanied by a fast and significant ontogenetic shift, in *Diplodus* spp. has not been sufficiently studied.

Here we investigate the origin of ecomorphological novelties in four *Diplodus* species*, D. vulgaris, D. sargus, D. puntazzo* and *D. annularis.* We argue that the ontogenetic niche shift is facilitated by changes in the developmental process and in turn these changes triggered the phenotypic evolution of these species. Due to the complex genetic basis of developmental processes, it is reasonable to assume that the ontogenetic pathways are highly conservative, and we expect that the examined species show little differences during their ontogeny. On the contrary, the alteration of the developmental process associated with significant morphological diversification could suggest an alteration of the developmental gene networks during the evolution of these species.

To test these hypotheses, we used a geometric morphometric (GMM) approach to investigate the pattern of shape variation across four ontogenetic stages in the four above-mentioned *Diplodus* species: the early, middle and late juvenile stages as well as the adult stage. This allowed us to have an insight in morphological development patterns across two ecologically and well differentiated life stages and to assess the process that ultimately lead to the adult shape. We assessed the degree of morphological differences among juveniles and adults between species, and we investigated the link between development and morphological differentiation by studying ontogenetic trajectories. These represent a useful formalization which allows investigation of the relationships between development and the evolution of complex phenotypes [23, 24]. Subsequently, we investigated the patterns of integration, modularity and disparity along the ontogeny. Integration and modularity are two tightly linked concepts. Integration is found when different traits have the tendency to vary jointly in the organism. Indeed, integration and modularity are a good proxy for the alteration of developmental process, which leaves traces in the pattern of variance and covariance [12]. Integration and modularity have become, in recent years, a central topic in evolutionary biology [25, 26]. A network of interactions is defined as modular if it is subdivided into relatively autonomous, internally highly connected components. Modules consist of parts that act together in the performance of some physiological function [25]. The strength of this interaction, e.g. the influence that each module “epigenetically” enacts on the other, may be different during the development of different taxa determining a different shape of the adult stage.

Our study represents a novel contribution to the understanding of the processes driving the evolution of inter-specific morphological differences among *Diplodus* species. It also offers the opportunity to shed more light on the role of development in triggering the evolution and appearance of novel phenotypic adaptations in species with complex life histories.

## Results

### Shape differences

**Fig. 1:**
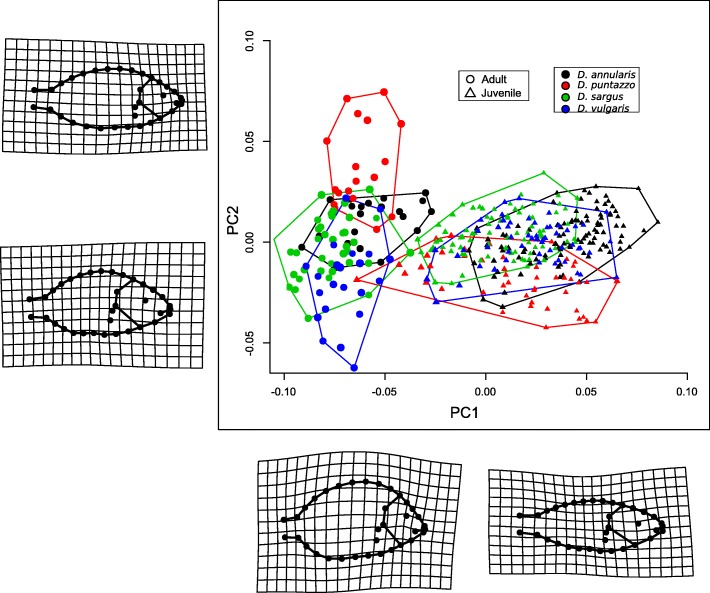
Principal Component Analysis of shape variables show the morphospace occupancy of the juveniles and adults of the four species (see the legend for symbols and colours significance). Each axis is associated with deformation grids (for positive and negative values) showing shape deformations. The first PC explain the 57.18% of the total variance whereas the second PC the 7.84%

Individuals are distributed along the first Principal Component (PC) axis according to their life stages (Fig. 1). Thus, PC1 represents mostly an axis summarizing shape changes during ontogeny. At positive PC1 scores we found juveniles belonging to all the four species. At negative PC1 scores we found only adults. On the other hand, PC2 mostly described the variation in the margin of caudal peduncle and in the widening of the opercular area which are modification more detectable among adult individuals. According to deformation grids (Fig. 1) juveniles (positive PC1 scores) show streamlined body shape and rounded heads. On the contrary, adults show a rounded body shape and a more pointed mouth. Within each life stage class, we found some overlap between species. There is no overlap between adults and juveniles of the same species, suggesting that the two life stages present well-differentiated shapes in all four species. According to Procrustes ANOVA differences among the four species between and within the two age classes are statistically significant (*p* < 0.001; Additional file 1: Table S1). The difference between developmental stages (juvenile-adult) largely exceed that between species (F_ls_ = 336.649, p < 0.001; F_species_ = 40.295, p < 0.001; Additional file 1: Table S1).

### Ontogenetic shape changes and disparity

Within each species, the model of simple multivariate allometry fits the data well but the bootstrap test for the directions of the growth trajectories reject the hypothesis of a common pattern of ontogenetic allometry (F_size*species_ = 21.941, p < 0.001; Additional file 1: Table S2). According to the test for homogeneity of slopes (Table 1) we found that *D. puntazzo* shows the largest angular difference of the multivariate allometric trajectory with respect to the other three species.

**Table 1:** Allometric slope comparisons

	*D. annularis*	*D. puntazzo*	*D. sargus*	*D. vulgaris*
*D. annularis*		0.001	0.008	0.001
*D. puntazzo*	15.431		0.001	0.001
*D. sargus*	3.714	39.016		0.001
*D. vulgaris*	6.955	47.585	8.578	

The diagonal pairwise effect size (Z-scores and the diagonal significance after 10,000 randomization are reported. These pairwise comparisons suggest a significant difference in the allometric vectors orientation, with *D. puntazzo* showing the highest divergence from other species

**Fig. 2:**
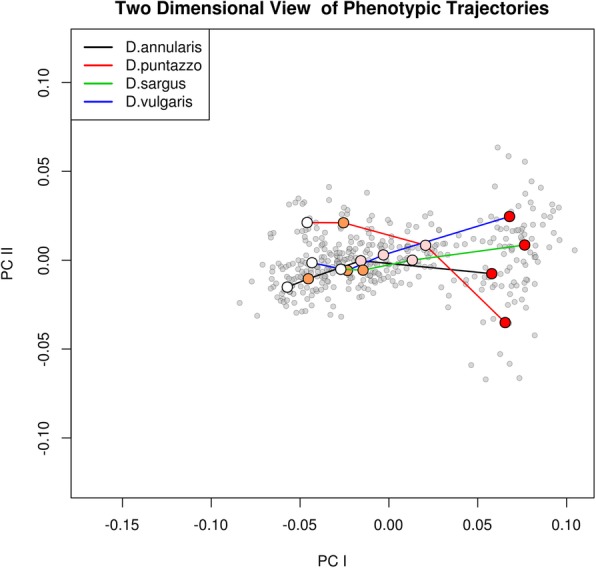
Ontogenetic trajectories, visualized in the space of principal components (PC1 vs. PC2), lining the four developmental stages *Diplodus* spp. Stage 1, 2 and 3 represent early settlement (white circle), middle settlement (orange circles) and late settlement (pink circle). Stage 4 includes only adult specimens (red circles). Juveniles converge on the same portion of the morphospace during the late settlement whereas trajectories show large differences when fishes recruit in the adult population

The phenotypic ontogenetic trajectories approach confirms the results on the ontogenetic allometry showing large and significant differences of the ontogenetic vectors angle in all the pairwise comparisons. Even in this case *D. puntazzo* shows the largest difference (Table 2). Furthermore, *D. puntazzo* is the only species showing significant differences in the shape of the ontogenetic trajectory whereas the other three species show similar directions of ontogenetic shape change across the four ontogenetic stages (Table 2). The difference of the shape of the phenotypic trajectory of *D. puntazzo* with respect to the other three species is clearly evident in Fig. 2 where juveniles *D. puntazzo* start from a different region of the morphospace occupied by juveniles of the four species in the early settlement (white circles) and end at an extreme of the morphospace occupied by adult specimens (red circles). One interesting feature of the observed inter-specific ontogenetic trends was the fact that at the end of settlement, the four species converge on a more similar shape (pink circles) respect to early settlement, suggesting a shape convergence during the development of juveniles. Then large differences are observed in the trajectory from late settlement to the adult stage.

**Table 2:** Differences in phenotypic trajectories among the four *Diplodus* species

	Θ	Z_Θ_	P_Θ_	D	Z_D_	P_D_
*D. annularis – D. puntazzo*	32.39	14.118	1.00E-04	0.176	2.941	0.0049
*D. annularis – D. sargus*	17.37	7.299	1.00E-04	0.079	0.288	0.3538
*D. annularis – D. vulgaris*	21.35	8.513	1.00E-04	0.103	0.675	0.238
*D. puntazzo – D. sargus*	36.89	17.675	1.00E-04	0.178	2.973	0.0051
*D. puntazzo – D. vulgaris*	43.67	19.343	1.00E-04	0.215	3.841	0.0006
*D. sargus – D. vulgaris*	14.16	4.707	1.00E-04	0.067	0.604	0.7068

Direction (θ = Pairwise Angles in degrees) and shape (D = Pairwise Procrustes Distance) differences of the four phenotypic trajectories across the four ontogenetic stages. The Z test value and the significance (P) obtained after 10,000 randomizations are reported for both direction and shape

**Fig. 3:**
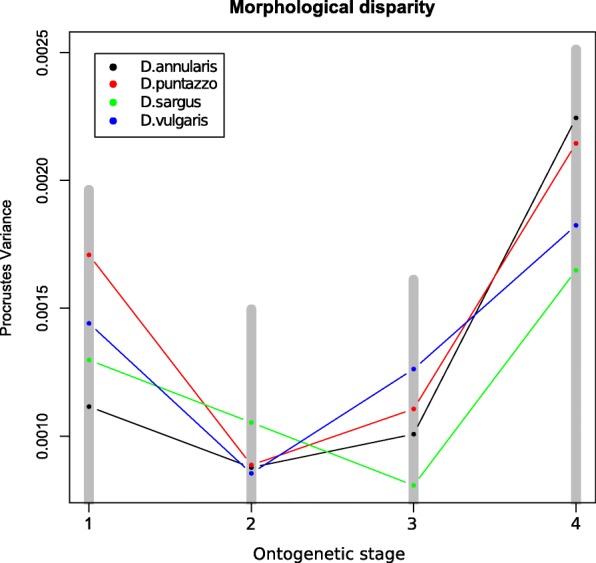
Morphological disparity across the four ontogenetic stages observed for each specimen. Lines represents the intraspecific morphological disparity for each species (see legend). The gray bars represent the inter-specific morphological disparity at each ontogenetic stage. Progressive ontogenetic stages are reported as number: 1 = early juveniles, 2 = middle juveniles, 3 = late juveniles, 4 = adults

The estimated ontogenetic morphological disparity shows a decreasing trend of morphological disparity (between and within species) of juveniles during settlement, followed by a drastic increase of disparity when the fishes reach the recruitment phase and become adults (Fig. 3). This pattern is constant across the four species and morphological disparity at the adult stage is always higher than that observed at the juvenile stage.

### Ontogenetic integration and modularity

**Fig. 4:**
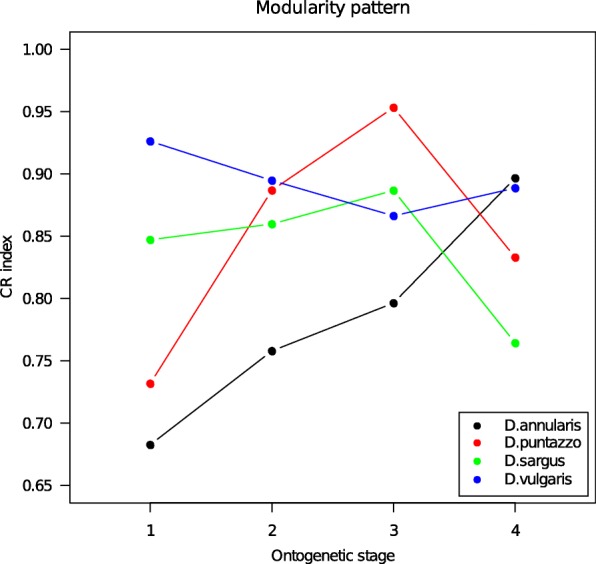
Pattern of modularity for the four species. Y axis represents the CR index. High values suggest low modularity (i.e. high covariance between modules). Progressive ontogenetic stages are reported as number: 1 = early juveniles, 2 = middle juveniles, 3 = late juveniles, 4 = adults

We estimated the CR index and the GI coefficient across the four ontogenetic stages for the four species. Modularity was significant at all stages, but we didn’t observe a common trend in the CR index between the four species (Fig. 4). For *D. annularis* and *D. puntazzo* the CR index suggests a lower modularity in juveniles at late settlement with respect to juveniles at early settlement. *D. sargus* and *D. vulgaris* CR show small changes during settlement.

Conversely, we observe marked changes in CR index during the shift from late settlement juveniles to adults. For *D. annularis*, the CR index still increases, suggesting a continuous decrease of modularity during ontogeny. *D. vulgaris* shows a coherent pattern with earlier ontogenetic stages with an almost invariant CR index and thus a low modularity preserved over all ontogeny. On the other hand, both *D. puntazzo* and *D. sargus* show a marked downshift of the CR index, suggesting an increase of modularity concurrently with recruitment.

**Fig. 5:**
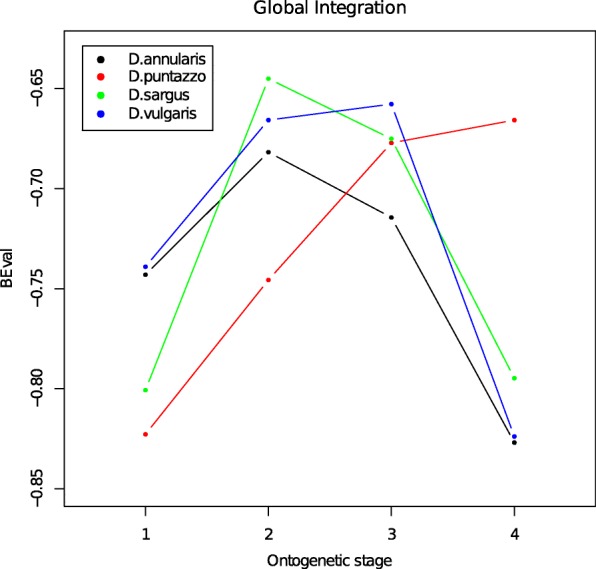
Global Integration pattern. The GI index describes the level of integration, self-similarity or disintegration in the four species (see legend). Progressive ontogenetic stages are reported as number: 1 = early juveniles, 2 = middle juveniles, 3 = late juveniles, 4 = adults

When we look at the GI coefficient (Fig. 5) we found a similar pattern among the four species during settlement. The GI always shows a deviation from a self-similar condition (coefficients larger than − 1). The GI coefficient tends to be less negative during growth. This disintegration pattern reflects localized shape changes associated with large variances of the corresponding PW. A large difference can be observed during the transition from juvenile at the late settlement to the adult life stage. Three species, *D. annularis*, *D. sargus* and *D. vulgaris*, show an increase of the GI coefficients through less negative values from late settlement juveniles to the adults, whereas *D. puntazzo* shows a continuous increase of GI trough a more dis-integrated condition.

## Discussion

During the earliest phase of their post-larval life individuals of the four *Diplodus* species already show species-specific shapes (Fig. 1) with a good degree of inter-specific morphological difference (Fig. 3). The fact that juveniles show interspecific differences is not new in *Diplodus spp*. Ventura et al. [19] have found head differences in juveniles *Diplodus* spp. and were able to link these differences to trophic habits. However, when juveniles and adults are analyzed together, the observed shape differences across life stages overwhelm those observed between species (Fig. 1; Additional file 1: Table S1). Juveniles show a rounded head shape and a streamlined body that makes juveniles of different species more similar than juveniles and adults of the same species (Fig. 1). On the other hand, adults generally show a larger inter-specific shape disparity however the inter-specific adult divergence is not so large as that observed between juveniles and adults of the same species (Additional file 1: Table S1). Frederich et al. [27, 28] recognized a similar pattern in Pomacentridae (damselfishes and clownfishes), explaining this fact as the result of a common selective pressure during different life stages. Different selective regimes would act on the two life stages suggesting that the observed morphological similarity among juveniles is the result of a strongly constrained developmental process. This constraint likely evolved to preserve an adaptive advantage in both juveniles and adults. Indeed, the juvenile shape is well suited for a pelagic life, allowing for the young fishes to move quickly and efficiently in the water column [29], which in turn reduces predation pressure.

What clearly emerged from our GMM analysis is that the juveniles of the four species become more similar as soon as they are approaching the recruitment phase (Fig. 2). Moreover, inter-specific disparity decreases during settlement in all four species, i.e. the phenotypic variability is constrained around the mean in all the four species and between species (Fig. 3). Shape convergence and phenotypic variance suppression are consistent with a developmental canalization pattern [30] during the juvenile stage, ending with juveniles of different species showing similar shapes. This would reinforce the idea that the juvenile shape develops under similar constraints in different species because they experience common selective pressures. Similarly, Kaufman et al. [31] hypothesize for Caribbean coral reef fishes that the functional convergence of morphology and coloration among transition juveniles can result from common selective pressures [31]. We could also speculate that the observed common shape features in *Diplodus* spp. late juveniles could also represent an advantageous morphology in preparation for the transition to a benthic environment. These would allow the settlers at the transition between juvenile and adults to feed on benthic environment while maintaining specializations that maximize survival in the pelagic environment. A similar pattern was hypothesized for the larval to juvenile transition (metamorphosis), a transition that would permit an individual that is well adapted to its present habitat to develop morphological characteristics necessary for the colonization of its next habitat [32, 33]. Additional data, including ontogenetic series from other fish with a bipartite lifestyle, will be necessary to confirm and to generalize the juvenile to adult transition pattern observed. Nonetheless, these observations pose interesting questions on the selective forces that triggered the evolution of a bipartite life history in *Diplodus* spp. We could speculate that if it is true that the shape convergence evolved to enhance the chance of survival of different species at the juvenile stage, this also likely produced a high inter-specific competition pressure. Thus, the evolution of diversified shapes in adults, representing a clear advantage to exploit different trophic resources, could be explained as character displacement in response to the inter-specific competition. It is interesting to note that *D. puntazzo*, a species showing a peculiar feeding ecology if compared with other *Diplodus* species, shows also the largest differences in term of ontogenetic trajectory and integration. This fact would suggest that natural selection could act selecting positively those alterations of ontogenetic trajectories that led to the appearance of morphological novelties that reduce interspecific competition.

As suggested by our analyses (Figs. 1 and 2) the shift from juvenile to adult shapes arises abruptly, allowing the fishes to quickly adapt to the new environment. We observed a drastic increase of disparity in adults (Fig. 3) and a remarkable shift of the phenotypic trajectories (Fig. 2) leading to the final adult shapes. These new adult phenotypes ensure better performances in a rocky benthonic environment [34, 35]. Few studies have investigated the mechanisms that trigger the ontogenetic shape shift between juvenile and adult fishes. Balon [36, 37] proposed a model of saltatory ontogeny which defines life stages as associations between ontogenetic changes in morphology and in habitat use. According to a saltatory model, development is not gradual but proceeds as a sequence of separate stable developmental states. The shape differences observed between juveniles and adult of *Diplodus* spp. in this study seem to fit well with a saltatory model and are in agreement with the life-history model for sparid fishes proposed by Vigliola & Harmelin-Vivien [38]. While the saltatory model describes well the shape changes we observe, it does not help understand how rapid ontogenetic shape shifts are guarantee in *Diplodus* spp., and more in general in fishes with a complex life cycle such as sparids. In other words, which mechanisms trigger the evolution of ontogenetic shape changes that are ultimately responsible for the shape of fishes with a bipartite life cycle? We suggest that the answer to this question can be found observing the intra and interspecific covariation patterns. Both the global integration and the ratio of within-between modules covariance follows a species-specific pattern over the four ontogenetic stages, suggesting that the development of the four species is characterized by different levels of interactions between traits and modules. We found that all the species shapes are globally disintegrated at all the ontogenetic stages, but at least in the early stages (early and middle juveniles), they share a reduction of landmark covariation. Modularity does not show a common pattern across the four species. This could suggest that the intensity of covariation in specific regions of the whole body changes between species and during ontogeny. The fact that the lower GI (except for *D. puntazzo*) is found concurrently with of intermediate developmental stages could suggest a major perturbation/change in developmental gene network regulation during these growth stages. However, at the same stages, we observe the smallest morphological disparity. In absence of genomic and transcriptomic analyses able to link morphological expression with its underlying genetic expression and regulation, we could only speculate that this could represent evidence of functional selective pressure toward a very specific morphology immediately preceding the adult stage. This means that the passage from pelagic to benthic ecology requires a specific morphology possibly linked to trophic niches and/or to hydrodynamic properties of body shapes irrespective of species-specific affiliation. The original concept of canalization was first proposed by Waddington [39]: “it describes the reduced sensitivity of a phenotype to genetic and environmental perturbations” (quoted in Salazar-Ciudad [40]) and, based on this definition, could imply a minor role of natural selection [40]. We argue that in the case of *Diplodus* species, the evidence of “diminished disparity” at the very particular point of passage between the two lifestyles cannot be explained by ignoring natural selection and functional morphology. Modularity and integration are good proxies for genetic network expression during development [41]. Trait covariation has its genetic basis mainly in pleiotropic genes able to affect more than one phenotypic characteristic [25, 42]. Modularity, which triggers the integration of certain traits and the decoupling of others, is supposed to define the increased evolvability of a phenotype, enhancing its capacity to evolve in response to selection [43]. This fact could suggest that the origin of morphological novelties is facilitated by different patterns of modularity and integration among species.

Changing integration and modularity during ontogeny allows *Diplodus* species to quickly modify their shape, converging on a similar shape in juveniles and allowing a quick shape divergence during the transition from juvenile to adult. Thus, both juvenile and adult phenotypes can be viewed as the results of the evolution of developmental patterns, and of the gene network behind them.

## Conclusions

Morphological development, reflecting complex gene networks tailored by evolution, could be expected to be highly conservative, especially in closely related species. Our results do not fit with this expectation, and we found that the evolution of advantageous shape changes in the four *Diplodus* species can be correlated with alterations in ontogenetic pathways.

The shape development is canalized in juveniles of the four species: they start from differentiated shapes and successively converge on the same portion of the morphospace. Conversely, we observe a clear divergence of ontogenetic trajectories, starting from late juveniles and producing highly divergent shapes of adults in the four species. The evolutionary significance of convergence and divergence observed at different stages of development poses interesting questions concerning the evolution of a bipartite lifestyle in demersal fish. Differential selective pressures can be claimed as the main driver of shape evolution in fishes with complex life cycles. Modularity and integration allowing the evolution of ontogenetic trajectories can be viewed as important sources of variability on which selection can act.

## Methods

### Study sites

**Fig. 6:**
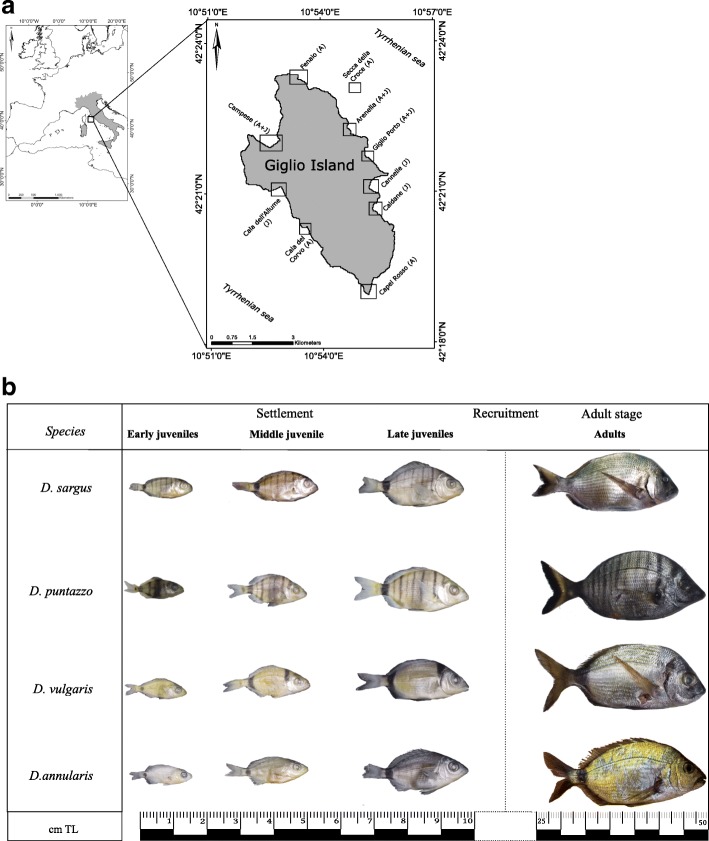
Study sites along the coast of Giglio Island is reported in Fig. 6a. In each site the letters in parenthesis indicate if juvenile (J), adults (A) or both (J-A) have been sampled. The map was drawn using the free and open source software Inkscape 0.91 (https://inkscape.org/) and online standard tile layer from OpenStreetMap data available at http://www.openstreetmap.org/. In Fig. 6b are reported the size classes collected in this study

The sampling campaigns were carried out along rocky coastlines of Giglio island, in the Central Tyrrhenian Sea (Fig. 6a). The coastal zone of this island is characterized by an high habitat heterogeneity, due to the co-occurrence of sandy bottoms with *Posidonia oceania* meadows and hard substrates with biogenic formation (e.g. coralligenous assemblages at depths greater than 30 m). Along these rocky granitic shorelines small coves and inlets are often present. In these sheltered sites the sea bottom exhibit a dense cover of photophilic algae on pebbly substrata and boulders which are suitable for the settlement of juvenile fish of the genus *Diplodus* [18, 34, 44].

Nursery areas in Giglio Island were also previously studied in Ventura et al. [22, 45]. To test the patterns of shape variation across ontogenetic stages, avoiding very spatially localized effects, we sampled both juvenile and adult fish, at 10 sites (i.e. both nursery grounds and adults fish habitats) along the whole coast of Island: Campese, Fenaio, Secca della Croce, Arenella, Giglio Porto, Cannelle, Caldane, Capel Rosso, Cala dell’Allume, Cala del Corvo (Fig. 6a). The localities are at least 2 km apart from each other.

### Sampling methods

Juvenile fishes were sampled with two hand nets (with a 2 mm mesh size) in nursery areas (sheltered shallow pebbly-rocky coves with gentle slope and sandy patches), whilst to obtain adult specimens we undertook spear fishing along abrupt rocky cliffs with large boulders. The sampling covered the bathymetric distribution from 0 to 40 m depth and was carried out during the whole year to match the months of presence of the juveniles (i.e. during their settlement periods: from May to July (*D. sargus*), from June to October (*D. annularis*), from November to February (*D. puntazzo*) and from February to May (*D. vulgaris*). Adult fishes were caught through spear fishing and juveniles were sampled with hand nets without using breathing apparatus. Major differences between juvenile and adult stages are reported in Fig. 6 b.

Juveniles were then anesthetized with low (160 mg/ml) concentrations of Tricaine methanesulfonate (Aqualife TMS, Syndel, Canada), also known as MS-222 (a muscle relaxant that blocks sodium and to a lesser degree potassium currents in nerve membranes). Subsequently, prolonged immersion (at least 10 min) with an overdose of MS-222 (200–300 mg/l) was used to euthanize fish.

A total of 390 individuals (291 juveniles and 99 adults) of the four species of the genus *Diplodus*, *D. sargus, D. puntazzo, D. vulgaris* and *D. annularis,* were collected (Table 3)*.* Juveniles refer to individuals collected during settlement, i.e. the time at which individuals change from the pelagic habit to the demersal habit typical of their juvenile and adult stages, from a standard length (SL) of 10 mm to a total length (TL) of 40–50 mm, depending on species (see Vigliola & Harmelin-Vivien [24]), up to the recruitment, i.e. the phase when the fish leaves the nursery area and reach the adult population. The juvenile stage was further divided into three size classes on the basis of the centroid size, i.e. the square root of summed squared distances between each landmark’s configuration and their centroid. These classes are: early, middle and late juveniles, in accordance with the classification based on fish developmental stages suggested in Vigliola & Harmelin-Vivien [38].

**Table 3:** Number of individuals sampled for each species (N total) during settlement and recruitment phases

	Settlement			Recruitment	N total
	early	middle	late	Adult	
*D. annularis*	34	35	37	21	127
*D. puntazzo*	16	15	17	18	66
*D. sargus*	26	27	29	38	120
*D. vulgaris*	18	18	19	22	77

Within settlement phase, juveniles were subdivided in three ontogenetic stages, early, middle and late, on the basis of the centroid size

The white sea bream *D. sargus* is abundant in the Mediterranean Sea and along the coast of South Africa [46, 47]. This species inhabits rocky bottoms and *Posidonia oceanica* beds to a depth of up to 50 m [18]. It is very active in the surf zone and feeds on seaweeds and benthic invertebrates [20]. The sharpsnout sea bream *D. puntazzo* is a benthopelagic marine species. Juveniles are gregarious and inhabit coastal waters (only occasionally over 50 m) on rocky or sandy bottoms. Adults often occur in the surf zone or on *P. oceanica* meadows and feed on seaweed, worms, mollusks and shrimps [48]. The two-banded sea bream *D. vulgaris* is distributed throughout the Mediterranean basin [46], on the Atlantic coast of the Iberian Peninsula and on the West African coast [49]. The juveniles are found in seagrass beds or on sandy areas whereas adults inhabits infra-littoral rocky bottoms more commonly up to a depth less than 50 m where they feed on benthic invertebrates [48]. The annular sea bream *D. annularis* is found in groups in sandy bottoms and seagrass beds, at depths ranging from 0 to 50 m. Juveniles are common on *P. oceanica* and *Zostera* spp. beds. It is a carnivorous species feeding on worms, crustaceans, mollusks, echinoderms and hydrozoans [48].

### Collected landmarks and modules definition

**Fig. 7:**
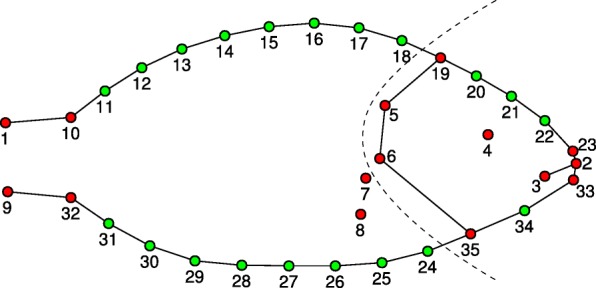
Landmarks collected on individuals: red points are fixed landmarks, green points are semilandmarks. Fixed landmarks were digitized on the most relevant and easily identifiable anatomical traits such as caudal peduncle, tips of the lips, eye and opercular area (see Loy et al. 2001 for a more exhaustive description). The dotted line shows the separation of the two modules considered in the analysis: head and trunk+tail

Thirty-five landmarks were collected on the whole body of 390 specimens (Fig. 7). Twenty landmarks, identified as points equally spaced along a segment starting and ending between two fixed landmarks (10–19, 19–23, 33–35, 32–35), were treated as semilandmarks [50, 51]. Semilandmarks allow the inclusion of curves that are considered anatomically homologous and discretizised by points in the shape analysis and can be analyzed together with fixed landmarks [38]. Landmarks were successively subdivided into two modules, the head and trunk+tail (Fig. 7), to perform specific analysis on integration and modularity (see below).

### Definition of ontogenetic stages

Sparids shows a complex life cycle with a sequence of ontogenetic stages associated with a different morphology and habitat use. Vigliola & Harmelin-Vivien [38] highlighted how successive ontogenetic changes occur in a specific order. During the settlement, a sequence of radical morphological changes allows the juveniles to shift from pelagic to benthic ecology but exact limits in size of stage shifts are difficult to estimate and are species-specific [38]. In order to have a better resolution of shape changes over ontogeny, juveniles collected during the settlement were split in three ontogenetic stages (Table 3 Fig. 6b) on the basis of the centroid size (CS): early settlement (from 1 to 33 CS percentile), middle settlement (from 34 to 66 CS percentile) and late settlement (> 67 CS percentile). Splitting individuals into different ontogenetic stages reduces the sample size. However, sample sizes are still robust enough to make inferences. Similar sample sizes were used in previous studies focusing on ontogeny of fishes [27, 28, 52].

#### Shape analysis

The shape analysis was performed using the R package geomorph [53]. The raw landmark configurations where aligned by using a Generalized Procrustes Analysis (GPA). Semilandmarks were allowed to slide so as to minimize shape the thin-plate spline bending. The aligned configurations obtained after the GPA were used to investigate shape differences and evolution through the juvenile and adult life stages. A Principal Component Analysis (PCA) was used to explore the multivariate morphospace and to establish shape variation within and among species. Statistical significance of the observed shape difference among species and age classes was assessed using a Procrustes ANOVA (10,000 permutations) with a residual randomization permutation procedure (sums of squares are calculated over many permutations to generate empirical probability distributions for evaluating model effects), a method well suited for multidimensional datasets [54]. Qualitative shape differences were visualized by producing thin-plate spline deformation grids.

#### Ontogenetic shape changes and disparity

Morphological differentiation during ontogeny was investigated by employing two different approaches. First, by using the function “procD.lm”, which performs Procrustes ANOVA with permutation procedures, we analyzed the ontogenetic allometry focusing on the relationship between size, here represented by the centroid size (CS), and shape. In this framework we assumed that the size variation is an indirect measure of the timing of the ontogenesis. The interaction between size and species effects was also evaluated in order to test for the occurrence of a common allometric trajectory. Subsequently a pairwise test for allometric slopes comparison was performed using the function “advanced.procD.lm” to quantify slope vector orientation (allometric trajectories) differences.

Successively the shape variability among four ontogenetic stages (Table 3) were investigated using a phenotypic trajectory analysis approach [55, 56]. In this approach it was possible to quantify the amount and the direction of shape changes (phenotypic trajectories) between different ontogenetic stages. The analysis was performed using the function “trajectory.analysis” that returns differences in angle, magnitude and shape of the trajectories (*P*-values estimated using 10,000 permutations).

To assess the degree of shape variability during ontogeny, the overall morphological disparity within and between species was calculated using the function “morphol.disparity”. This function estimates the morphological disparity as the Procrustes variance, calculated as the sum of the diagonal elements of the group covariance matrix divided by the number of observations in the group [57]. The statistical significance of the observed differences was assessed through permutations (10,000 randomizations).

#### Integration and modularity

Integration and modularity are two tightly linked concepts. Integration is found when different traits have the tendency to vary jointly in the organism. On the other hand, an organism is modular if its traits (landmarks in GMM) can be divided into two or more sets characterized by a strong within module integration and a relative weak covariance between modules [26].

Different indexes have been proposed to measure integration and modularity. Here we used the covariance ratio (CR) index [58] to evaluate modularity and the Global Integration index (GI) recently proposed by Bookstein [59] to measure the degree of integration. Integration and modularity were evaluated for each species at the four ontogenetic stages. The CR index is simply a ratio between the covariance within and between modules that are relatively autonomous with respect to each other. Significant modularity is found when the CR coefficient is small relative to a null distribution, which is centered on 1. Specifically, CR values between 0 and 1 describe datasets where the degree of covariation between modules is less than that found within modules, which characterizes relatively more modular structures. By contrast, CR values larger than 1 describe greater covariation between modules relative to within modules which characterizes less modular structures. The global integration (GI) coefficient quantifies integration across the entire organism. In the GI approach, the natural logarithm of bending energies (BEs, i.e. the eigenvalues of the bending energy matrix computed on the Procrustes consensus are regressed against the logarithm of variances of the corresponding partial warps (PW, i.e. the collection of projections of each aligned configuration on the eigenvectors of the above mentioned bending energy matrix). This relationship indicates how the variance of PW decreases when the localization of the corresponding deformation (represented by BEs) increases. As the self-similarity is claimed to occur approximately at the beta regression value of − 1 (in the log-log relationship) [59], the resulting slope could suggest integration when it is smaller than − 1 or disintegration when it is larger than − 1).

## Additional file

Additional file 1: **Table S1.** Procrustes ANOVA test for the significance of the species, life stage effects and their interaction on shape differences; **Table S2.** Test for a common multivariate allometric trajectory. (PDF 17 kb)

## Abbreviations

BE: Bending energy
CR: Covariance ratio
CS: Centroid size
GI: Global Integration
GMM: Geometric Morphometrics
GPA: Generalized Procrustes Analysis
PC: Principal Component
PCA: Principal Component Analysis
PW: Partial warps
SL: Standard length
TL: Total length

## References

[1] Darwin C (1859). On the origin of species by means of natural selection.

[2] Dobzhansky T (1937). Genetics and the origin of species.

[3] Orr HA (1995). The population genetics of speciation: the evolution of hybrid incompatibilities. Genetics..

[4] Orr HA, Orr LH (1996). Waiting for speciation: the effect of population subdivision on the time to speciation. Evolution..

[5] Fusco G (2001). How many processes are responsible for phenotypic evolution?. Evol Dev..

[6] Balon EK (2002). Epigenetic processes, when Natura non Facit Saltum becomes a myth, and alternative ontogenies a mechanism of evolution. Environ Biol Fish.

[7] Alberch P (1980). Ontogenesis and Morphological Diversification. Am Zool.

[8] Müller GB (2007). Evo–devo: extending the evolutionary synthesis. Nat Rev Genet..

[9] Moczek AP (2008). On the origins of novelty in development and evolution. BioEssays..

[10] Porter AH, Johnson NA (2002). Speciation despite gene flow when developmental pathways evolve. Evolution..

[11] Evans KM, Waltz B, Tagliacollo V, Chakrabarty P, Albert JS (2017). Why the short face? Developmental disintegration of the neurocranium drives convergent evolution in neotropical electric fishes. Ecol Evol.

[12] Klingenberg CP, Hallgrímsson B, Hall BK (2005). Developmental constraints, modules, and Evolvability. Variation.

[13] Zelditch ML, Sheets HD, Fink WL (2003). The ontogenetic dynamics of shape disparity. Paleobiology..

[14] Klingenberg CP (2010). Evolution and development of shape: integrating quantitative approaches. Nat Rev Genet..

[15] Voje KL, Hansen TF, Egset CK, Bolstad GH, Pélabon C (2014). Allometric constraints and the evolution of allometry. Evolution..

[16] Pélabon Christophe, Firmat Cyril, Bolstad Geir H., Voje Kjetil L., Houle David, Cassara Jason, Rouzic Arnaud Le, Hansen Thomas F. (2014). Evolution of morphological allometry. Annals of the New York Academy of Sciences.

[17] Hallgrímsson B, Lieberman DE, Liu W, Ford-Hutchinson AF, Jirik FR (2007). Epigenetic interactions and the structure of phenotypic variation in the cranium. Evol Dev.

[18] Harmelin-Vivien ML, Harmelin JG, Leboulleux V (1995). Microhabitat requirements for settlement of juvenile sparid fishes on Mediterranean rocky shores. Hydrobiologia..

[19] Ventura D, Bonhomme V, Colangelo P, Bonifazi A, Lasinio GJ, Ardizzone G (2017). Does morphology predict trophic niche differentiation? Relationship between feeding habits and body shape in four co-occurring juvenile species (Pisces: Perciformes, Sparidae). Estuar Coast Shelf Sci.

[20] Sala E, Ballesteros E (1997). Partitioning of space and food resources by three fish of the genus Diplodus (Sparidae) in a Mediterranean rocky infralittoral ecosystem. Mar Ecol Prog Ser.

[21] Costa C, Cataudella S (2007). Relationship between shape and trophic ecology of selected species of Sparids of the Caprolace coastal lagoon (Central Tyrrhenian sea). Environ Biol Fish.

[22] Ventura D, Jona Lasinio G, Ardizzone G (2015). Temporal partitioning of microhabitat use among four juvenile fish species of the genus Diplodus (Pisces: Perciformes, Sparidae). Mar Ecol.

[23] Alberch P, Gould SJ, Oster GF, Wake DB (1979). Size and shape in ontogeny and phylogeny. Paleobiology..

[24] Klingenberg CP (1998). Heterochrony and allometry: the analysis of evolutionary change in ontogeny. Biol Rev.

[25] Wagner GP, Pavlicev M, Cheverud JM (2007). The road to modularity. Nat Rev Genet..

[26] Klingenberg CP (2008). Morphological integration and developmental modularity. Annu Rev Ecol Evol Syst.

[27] Frédérich B, Vandewalle P (2011). Bipartite life cycle of coral reef fishes promotes increasing shape disparity of the head skeleton during ontogeny: an example from damselfishes (Pomacentridae). BMC Evol Biol.

[28] FREDERICH BRUNO, ADRIAENS DOMINIQUE, VANDEWALLE PIERRE (2008). Ontogenetic shape changes in Pomacentridae (Teleostei, Perciformes) and their relationships with feeding strategies: a geometric morphometric approach. Biological Journal of the Linnean Society.

[29] Müller UK, Videler JJ (2004). Inertia as a ‘safe harbour’: do fish larvae increase length growth to escape viscous drag?. Rev Fish Biol Fish.

[30] Hallgrímsson B, Willmore K, Hall BK (2002). Canalization, developmental stability, and morphological integration in primate limbs. Am J Phys Anthropol.

[31] Kaufman L, Ebersole J, Beets J, McIvor CC (1992). A key phase in the recruitment dynamics of coral reef fishes: post-settlement transition. Environ Biol Fish.

[32] Lough R, Pennington M, Bolz G, Rosenberg A (1982). Age and growth of larval Atlantic herring, Clupea harengus L, in the gulf of Maine-Georges Bank region based on otolith. Growth increments. Fish Bull.

[33] Copp GH, Kovác V (1996). When do fish with indirect development become juveniles?. Can J Fish Aquat Sci.

[34] Loy A, Bertelletti M, Costa C, Ferlin L, Cataudella S (2001). Shape changes and growth trajectories in the early stages of three species of the genus Diplodus (Perciformes, Sparidae). J Morphol.

[35] Weihs D (1986). Functional locomotor morphology of early life history stages of fishes AU – Webb, Paul W. Trans Am Fish Soc.

[36] Balon EK (1975). Terminology of intervals in fish development. J Fish Res Board Can.

[37] Balon EK (1990). Epigenesis of an epigeneticist: the development of some alternative concepts on the early ontogeny and evolution of fishes. Guelph Ichthyol Rev.

[38] Vigliola L, Harmelin-Vivien M (2001). Post-settlement ontogeny in three Mediterranean reef fish species of the genus Diplodus. Bull Mar Sci.

[39] Waddington CH. The strategy of the genes. Allen & Unwin; 1957.

[40] Salazar-Ciudad I (2007). On the origins of morphological variation, canalization, robustness, and evolvability. Integr Comp Biol.

[41] Melo D, Porto A, Cheverud JM, Marroig G (2016). Modularity: genes, development, and evolution. Annu Rev Ecol Evol Syst.

[42] Wagner GP, Zhang J (2011). The pleiotropic structure of the genotype–phenotype map: the evolvability of complex organisms. Nat Rev Genet.

[43] Hansen TF (2003). Is modularity necessary for evolvability?: remarks on the relationship between pleiotropy and evolvability. Biosystems..

[44] García-Rubies A, Macpherson E (1995). Substrate use and temporal pattern of recruitment in juvenile fishes of the Mediterranean littoral. Mar Biol.

[45] Ventura D, Bruno M, Lasinio GJ, Belluscio A, Ardizzone G (2016). A low-cost drone based application for identifying and mapping of coastal fish nursery grounds. Estuar Coast Shelf Sci.

[46] Tortonese E (1975). Osteichthyes (pesci ossei). Fauna d’Italia.

[47] Christensen M (1978). Trophic relationships in juveniles of three species of sparid fishes in the south African marine littoral. Fish Bull.

[48] Bauchot M, Hureau J (1986). Sparidae. Fishes of the north-eastern Atlantic and Mediterranean.

[49] De La Paz R. Systématique et phylogenèse des Sparidae du genre Diplodus Raf., (Pisces, Teleostei). ORSTOM; 1975. http://www.documentation.ird.fr/hor/fdi:07643.

[50] Bookstein F. Morphometric tools for landmark data: geometry and biology. 1991.

[51] Gunz P, Mitteroecker P (2013). Semilandmarks: a method for quantifying curves and surfaces. Hystrix Ital J Mammal..

[52] Frédérich B, Colleye O, Lepoint G, Lecchini D (2012). Mismatch between shape changes and ecological shifts during the post-settlement growth of the surgeonfish, *Acanthurus triostegus*. Front Zool.

[53] Adams DC, Otárola-Castillo E (2013). Geomorph: an r package for the collection and analysis of geometric morphometric shape data. Methods Ecol Evol.

[54] Collyer ML, Sekora DJ, Adams DC (2014). A method for analysis of phenotypic change for phenotypes described by high-dimensional data. Heredity..

[55] Adams DC, Collyer ML (2009). A general framework for the analysis of phenotypic trajectories in evolutionary studies. Evolution..

[56] Collyer ML, Adams DC (2013). Phenotypic trajectory analysis: comparison of shape change patterns in evolution and ecology. Hystrix Ital J Mammal.

[57] Zelditch ML, Swiderski DL, Sheets HD, Fink WL, Zelditch ML, Swiderski DL, Sheets HD, Fink WL (2004). 12 - disparity and variation. Geometric Morphometrics for biologists.

[58] Adams DC (2016). Evaluating modularity in morphometric data: challenges with the RV coefficient and a new test measure. Methods Ecol Evol.

[59] Bookstein FL (2015). Integration, disintegration, and self-similarity: characterizing the scales of shape variation in landmark data. Evol Biol.

