# Supplementary material for: Are developmental shifts the main driver of phenotypic evolution in Diplodus spp. (Perciformes: Sparidae)?
Source: BMC Evol Biol. 2019 May 21;19:106. doi: 10.1186/s12862-019-1424-1 (PMC6528360; doi:10.1186/s12862-019-1424-1)
Supplement: Supplementary file 1 — Table S1. Procrustes ANOVA test for the significance of the species, life stage effects and their interaction on shape differences; Table S2. Test for a common multivariate allometric trajectory. (PDF 17 kb) [file 12862_2019_1424_MOESM1_ESM.pdf]

Supplementary table 1: Procrustes ANOVA. The significance of the effect of species, life stage (ls) and their interaction on shape differences was tested. For each effect the degree of freedom (Df) the sum of squares (SS), the mean squares (MS), the r squared (Rsq), the F and Z test values and significance (P) after 10000 randomizations are reported.

	Df	SS	MS	Rsq	F	Z	P
Species	3	0.20563	0.06854	0.13742	40.295	8.5478	1e-04
ls	1	0.57264	0.57264	0.38270	336.649	8.9929	1e-04
Species:ls	3	0.06826	0.02275	0.04562	13.377	11.6155	1e-04
Residuals	382	0.64979	0.00170				
Total	389	1.49632					

Supplementary table 2: Test for a common multivariate allometric trajectory. The significance of the effect of size, species and their interaction on shape differences was tested. The presence of significant interaction between species and size effect suggest the lack of a common multivariate allometric trajectory. For each effect are reported the degree of freedom (Df) the sum of squares (SS), the mean squares (MS), the r squared (Rsq), the F and Z test values and significance (P) after 10,000 randomization.

	Df	SS	MS	Rsq	F	Z	Pr(>F)
size	1	0.56788	0.56788	0.37952	318.022	8.8164	1e-04
species	3	0.12876	0.04292	0.08605	24.037	12.0262	1e-04
size*species	3	0.11754	0.03918	0.07855	21.941	11.9186	1e-04
Residuals	382	0.68213	0.00179				
Total	389	1.49632					
